# Motion Aftereffects From Moving Illusions

**DOI:** 10.1177/2041669518811305

**Published:** 2018-12-03

**Authors:** Stuart Anstis

**Affiliations:** Department of Psychology, University of California, San Diego, La Jolla, CA, USA

**Keywords:** motion, aftereffect, illusion

## Abstract

Lines in the café wall illusion, and motion trajectories in the furrow illusion, appear to be tilted away from their true orientations. We adapted to moving versions of both illusions and found that the resulting motion aftereffects were appropriate to their perceptual, not their physical, orientations.

## Introduction

We examined two geometrical illusions, one normally static and the other one moving. In the static *café wall illusion* (Gregory, 1979; Münsterberg, 1897), horizontal lines appear to be tilted away from the horizontal. In the moving *furrow illusion* (Anstis, 2012), the vertical trajectory of a downward-moving spot appears to be tilted away from the vertical. We adapted to a moving version of the café wall illusion and then stopped the motion. This typically yields a motion aftereffect (MAE) in a direction opposite to the adapting motion (reviewed by Anstis, Verstraten, & Mather, 1998; Mather, Verstraten, & Anstis, 1998). Again, we adapted to moving spots in the furrow illusion and then stopped the motion. Was the MAE appropriate to the physical or the perceptual orientation of the moving lines and spots, respectively?

In the café wall illusion, straight, parallel horizontal dividing lines between staggered rows of alternating black and white *bricks* appear to be sloping or tilted. It was first described by Münsterberg (1897) and rediscovered by Gregory and Heard (1979), who used mid-gray lines of *mortar* between the black and white bricks. See also Kitaoka, Pinna, and Brelstaff (2001, 2004).

Movie 1 shows a moving version of the café wall illusion. In the first frame, the straight, parallel horizontal lines just above and below the fixation point look like a long, shallow horizontal V, and they appear to be closer together on the right than on the left. For the first 500 milliseconds, the café wall pattern moves to the left, and these two lines appear to move closer together. For the next 500 milliseconds, the whole pattern is left–right mirror-reversed, and it also reverses direction and moves back to the right. This also makes the same two central lines appear to move closer together. This sequence repeats indefinitely.

**Movie 1.** (click to play). Fixate the red spot for ∼30 seconds, then stop the movie yourself. Note the vertical MAEs of the horizontal gray lines, even though these never moved across the retina.

Following 30 seconds of steady fixation, the back-and-forth motion was stopped. Veridical perception ought to yield no aftereffect of motion because we carefully kept the gray lines stationary and horizontal, with no vertical motion (and the back-and-forth horizontal motions of the bricks would cancel out). But we found a strong aftereffect of motion, when the motion was stopped the horizontal lines immediately above and below the fixation point appeared to expand outward. The two more peripheral rows, whose illusory slope was in the opposite direction, appeared to contract slightly inward during the MAE. An adapting speed of 10 deg/s gave an MAE that could be approximately matched by short horizontal lines (not shown) that moved apart vertically at 5 arcmin/s. Adapting speeds of 5 deg/s or 20 deg/s gave reduced MAEs.

Morgan and Moulden (1986) attributed the Münsterberg or café wall tilt effect to circularly symmetric visual band-pass filtering, which produces peaks and troughs along the mortar that are oriented along lines slightly tilted with respect to the horizontal. The result is a Fraser twisted cord, which underlies the Münsterberg effect.

The MAE shows that the neural representations of the horizontal lines must include tilted components (consistent with Morgan and Moulden’s model) that can stimulate and adapt, low-level *short-range motion* detectors of limited spatial range (Braddick, 1974),

An entirely different illusion is the dynamic furrow illusion (Anstis, 2012). When a spot drifts vertically downward across a field of 45° oblique parallel stripes, the motion is seen veridically in foveal vision, but in peripheral vision, its path seems strongly oblique, often becoming almost parallel to the background stripes. Note that the spot path is attracted toward the orientation of the background stripes not repelled away from it; unlike most geometrical illusions, this is an illusion of orientation assimilation not orientation contrast.

Following adaptation to a spot that moved downward across a tilted grating, was the MAE upward, opposite to the spot’s physical motion, or oblique, opposite to its perceived motion?

Two parallel, vertical columns of 0.5° diameter red dots were spaced 5° apart horizontally and 1.67° apart vertically. They drifted downwards at 5 deg/s, across a static background of stripes of 1.27 cycles per degree that were tilted −45° and +45° from the vertical to form parallel V-chevrons, as shown in Movie 2.

**Movie 2.** (click to play). Fixate the black spot. The peripheral red spots actually move vertically downward but appear to converge toward the middle. The MAEs seen in the static blue spots appear to diverge outward, approximately opposite to the *perceived* (not physical) motion. Blue and red arrows show the mean settings of five observers.

Following 12 seconds of motion, the motion was stopped for 3 seconds, and the dots turned blue for easier identification. (Color labels did not alter the motion.) This adapt-test sequence recycled indefinitely. Observers fixated on a point located 10° to the right of the stimulus and struck keys to adjust the orientation of two pairs of arrows (red for adapting and blue for MAE) that stuck out from the fixation point.

Given veridical perception, observers would have adjusted the red and blue arrows to point down and up, respectively. But in fact, observers set the red (adapting) arrows to converge at angles of ± 52.5° away from the vertical, and they set the blue (MAE) arrows to diverge at angles of ± 64° away from the vertical. (We attribute this 11.5° difference to experimental error.)

## Conclusion

The MAE was appropriate to the *perceived* not the physical trajectory of the moving spots. So the furrow illusion must stimulate low-level motion detectors (Braddick, 1974) and arise before the MAE, which probably reflects activity in middle temporal area (V5; Tootell et al., 1995). Just as the neural representation of the café wall contours must contain some oriented components, so must the furrow trajectories. Our results ascribe both illusions to early, bottom-up physiology not to top-down cognition.
